# Association of Connective Tissue Grafts in Immediate Implants: Systematic Review and Meta-Analysis

**DOI:** 10.3390/dj12060183

**Published:** 2024-06-17

**Authors:** Marta Torra-Moneny, Elisabet Mauri-Obradors, Sonia Egido-Moreno, Joan Valls-Roca-Umbert, Antonio Marí-Roig, José López-López

**Affiliations:** 1 Department of Odontostomatology, Faculty of Medicine and Health Sciences (Dentistry), University of Barcelona, 08907 Barcelona, Spain; mtorramon9@alumnes.ub.edu (M.T.-M.); lismauri3@gmail.com (E.M.-O.); joan.valls@yahoo.es (J.V.-R.-U.); amari@bellvitgehospital.cat (A.M.-R.); 2 Maxillofacial Surgery Service, Bellvitge University Hospital, 08907 Barcelona, Spain; 3 Oral Health and Masticatory System Group, Institut d’Investigació Biomédica de Bellvitge IDIBELL (Bellvitge Institute of Biomedical Research), 08907 Barcelona, Spain; 4 Medical Surgical Area of the Dental Hospital, University of Barcelona (HOUB), 08907 Barcelona, Spain

**Keywords:** dental implant, dental implantation, implant, single tooth dental, immediate dental implant loading, connective tissue

## Abstract

Background: The increase in soft tissue (ST) around implants can benefit peri-implant health and aesthetic results. The objective was to compare the gingival and esthetic health benefits of immediate implant placement (IIP) with simultaneous or delayed connective tissue graft (CTG) compared to IIP without CTG. Methods: A systematic review was carried out by two reviewers in Medline-Pubmed, Scopus, and Cochrane. The Preferred Reporting Items for Systematic Reviews and Meta-Analyses (PRISMA) were considered. Randomized Clinical Trials (RCTs) that were published between April 2017 and February 2024 were used. Studies that analyzed the performance of a simultaneous or deferred CTG after the placement of an implant in the aesthetic zone, with or without immediate provisionalization, without previous regeneration, with a follow-up of 6 months, and that were performed in humans were included. Results: Quantitative analysis was performed using data provided by the RCTs. The five RCTs that were selected analyzed a total “*n*” of 245 subjects who met the inclusion criteria and focused on the subject of the study. In the quantitative analysis, four RCTs were included. The studies evaluated buccal gingiva levels when placing the IIP with and without CTG, obtaining a mean buccal gingiva level difference of 0.09 mm (95% CI: −0.54 to 0.72, *p* = 0.05), statistically not significant, but with a favorable trend. Conclusions: The use of CTG associated with the II can maintain the gum level but not increase the volume. CTG is favorable for achieving successful esthetic results when immediate placement of an implant with a provisional prosthesis is planned.

## 1. Introduction

The concept of gingival biotype was introduced in 1991 when it was stated that a minimum keratinized gingiva in width and thickness was required around the teeth [1]. Furthermore, with the advancement of implantology, the importance of soft tissue (ST) has been increasing [2].

A review carried out by Thoma et al. [3] concluded that ST augmentation results in less recession of the mid-buccal mucosa and increased mucosa thickness. The union of ST around the implant is considered a biological seal that prevents peri-implant diseases [2]; this disease is characterized by a greater accumulation of plaque, bleeding on probing, and ST recession [4]. ST augmentation around implants may be beneficial for peri-implant health and preventing peri-implant disease since implants that receive ST augmentation are likely to have less marginal bone loss [5]. This procedure can be carried out simultaneously when placing the implant or in a deferred manner [6]; nevertheless, there are controversies regarding the results, and a recommendation cannot be given on the preferable time point for soft tissue augmentation [3,7,8].

There have been several techniques and materials proposed to increase soft tissue around implants. Numerous biomaterials have been proposed and are constantly in development. Allografts are substitute grafts extracted from a subject of the same species. The advantages are osteoconduction, osteoinduction, and availability, while the limitations are biocompatibility or possible transmission of diseases. Xenografts are most commonly used and diffused for oral regeneration owing to their osteoconduction properties. However, xenografts are commonly harvested from other species and usually derive from bovine, porcine, or equine origin. In addition, another source of xenografts is represented by marine substitutes (e.g., coral skeletons, fish bones, etc.), which show potential osteoconduction properties beneficial to periodontal and bone regeneration [9].

The “gold standard” and the aim of our review for ST augmentation is the own autologous connective tissue graft (CTG); the advantages are biocompatibility, osteoinduction, and regenerative potential. This can be obtained from palate or tuberosity [5], providing an increase in the amount of queratinized gingiva that may prevent inflammatory complications or unsatisfactory esthetic results [10]. Aesthetic demand of patients has increased over time [11]. A harmony of peri-implant soft and hard tissues is required to achieve this, as these will be essential for optimal function and aesthetics [6]. As an alternative, the use of a xenogeneic collagen matrix or acellular dermal matrix could be used to reduce patient morbidity, instead of autologous connective tissue [12]. However, according to Cairo F et al. [13], better results are obtained with the application of autologous CTG. Puisys A et al. [14] referred to similar aesthetic results between CTG and the collagen matrix.

It is known that immediate implant placement (IIP) does not prevent the loss of hard or soft tissue volumes that occur after extraction [15], but alveolar bone atrophy after a tooth extraction is far less severe when immediate implantation is performed [16] and reduce treatment time [17]. It also increases patient acceptance for implant treatment. IIP provides benefits for all the available bone and soft tissues, which tend to decrease in volume for delayed or late implants. The depth, angulation, and distance from the adjacent teeth or implants determine the future stability of the peri-implant bone and soft tissues and, thus, the overall success and implant survival. Cone beam computed tomography (CBCT), with dedicated software, is an excellent tool for evaluating the available bone and plan for optimal implant shape and position [16].

ST contraction can compromise the aesthetic results of an implant-supported single-unit restoration due to the changes that occur in the gingiva, especially in the upper jaw [15]. Furthermore, the lack of thickness of the alveolus’s vestibular wall, the implant’s inadequate position, and a thin gingival biotype are factors related to the loss of volume of these tissues [15,18]. A combination of immediate implant placement, connective tissue graft, and early implant placement tends to result in less soft tissue regression due to the thicker postoperative facial soft tissue volume preserved [19].

A non-functional immediate provisional restoration and a CTG or a flapless approach are options for preventing vestibular recession of the area treated with an IIP [15]. According to the literature, in 11% of the cases of immediate provisionalization, there is a recession (≥1 mm) of the vestibular gingiva that continues until 5 years after implant placement [17]. Some authors have suggested the placement of implants alongside CTG [20]. Furthermore, the use of customized healing abutments has been proposed since a better trend in preservation of peri-implant soft tissue, esthetic outcomes, and lower patient discomfort in immediate implant sites has been demonstrated. This use of customized healing abutments has shown the preservation of peri-implant buccal horizontal soft tissue and buccal volume and the preservation of the papilla height and midfacial height [21].

Although CTG is essential for gingival health, there is no clear agreement, which is why we propose this review. The aim of this systematic review and meta-analysis was to compare the gingival and esthetic health benefits of IIP with simultaneous or delayed CTG compared with IIP without CTG.

## 2. Materials and Methods

To carry out this systematic review, we used the Preferred Reporting Items for Systematic Reviews and Meta-Analyses (PRISMA) [22]. A detailed protocol was prepared before starting the review and registered on Prospero (CRD42024515781).

The following PICO question was posed: (P) Population: patients who have a tooth to be extracted in the aesthetic sector and who received an IIP. (I) Intervention: patients requiring simultaneous or delayed CTG. (C) Comparison: patients without CTG. (O) Outcome: evaluate the benefits and disadvantages of performing a CTG during the first phase of surgery or in the second phase or delayed surgery after IIP by analyzing the buccal gingival level.

A bibliographic search was carried out in electronic databases of Medline-Pubmed, Scopus, and Cochrane. The search used the keywords “dental implant” OR “dental implantation” OR “implant, single tooth dental” OR “immediate dental implant loading” AND “connective tissue” OR “soft tissue augmentation” OR “sub-epithelial connective tissue graft” combined with the Boolean terms. The inclusion criteria were articles that analyzed the performance of a simultaneous or deferred CTG after the placement of an II in the esthetic zone, with or without IIP, without prior regeneration in the area where the implant was placed, with a follow-up period of at least of the cases of 6 months, Randomized Clinical Trials (RCTs) that were published between April 2017 and February 2024, which were carried out in humans.

Studies that were not RCTs, that did not treat in the aesthetic sector together with CTG, with samples of less than 24 patients because they are not so representative, and where implants were placed in regenerated bone were excluded.

The JADAD scale was used to assess methodological quality [23], and the Oxford Classification of Evidence Levels (OCEBM) was used to assess the scientific evidence of clinical trials [24]. The assessment of the risk of bias of each included RCT was performed using version 1 of the Cochrane risk-of-bias tool for RCTs (RoB1). RoB1 individual domains address the following seven types of bias categorized as follows: (1) Low risk of bias if all domains were at low risk; (2) Unclear risk of bias if one or more domains were unclear risk; or (3) High risk of bias if ≥1 domains were at high risk. The review was carried out by M.T.-M and E.M.-O, and in case of discrepancy, J.L.-L was consulted.

### Statistical Analysis

To perform the meta-analysis, the common variables of the different studies included in the review were analyzed. The Review Manager 5.4^®^ program was the tool used to perform the statistical analysis. Forest plots were performed to graphically represent the difference between the values studied in the articles regarding the placement of a CTG or not, reported with a confidence interval (CI) of 95%. For the level of significance, a *p*-value (*p*) = 0.05 was used. Heterogeneity was assessed using the I^2^ statistic. Heterogeneity among studies was considered statistically significant for a *p*-value < 0.05 and was interpreted as recommended by the Cochrane Handbook: 0–40% was considered unimportant, 30–60% as moderate heterogeneity, 50–90% as substantial heterogeneity, and 75–100% as considerable heterogeneity.

## 3. Results

**Figure 1:**
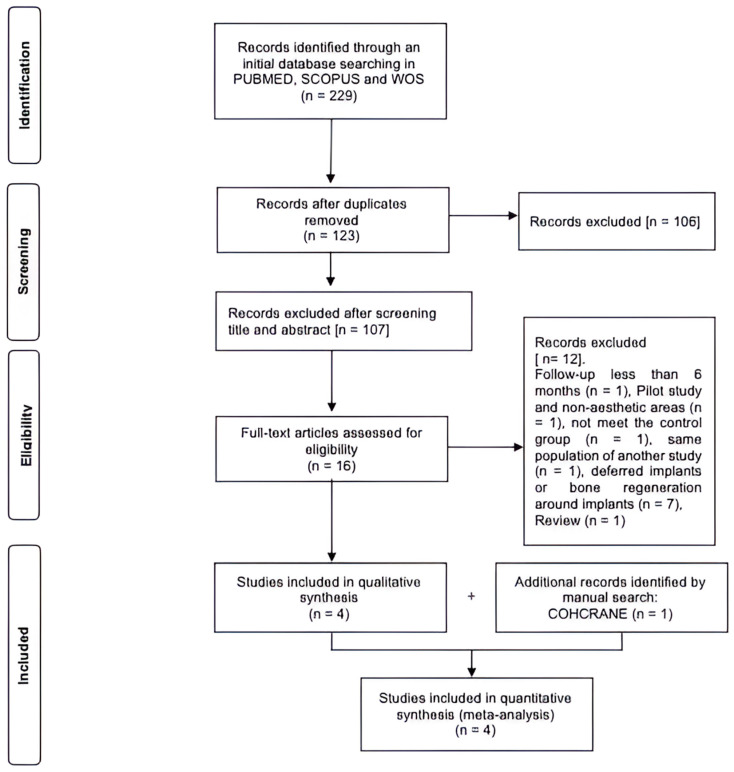
Flow chart illustrating the search strategy.

With the electronic search, a total of 229 articles were found in the Pubmed, Scopus, or Web of Science (WOS) databases using the keywords combined with the Boolean terms AND and OR. From this search, the duplicates were removed, and titles and abstracts of all the papers were read, resulting in 16 articles being selected. Twelve articles were excluded due to the following reasons: one article because it was a review [25], one article [26] because it followed up the cases for <6 months, another [4] because it was a pilot study and included II in non-aesthetic areas, another [27] because it did not meet the control group (CG) criteria without graft, another [18] because it used the same population of another included RCT [28], six articles [2,5,12,20,26,29] because they included deferred implants, and/or five more articles [11,12,29,30,31] because they performed bone regeneration in the area where the implants were placed. Four articles were selected, and one article from the Cochrane database was added. Finally, five RCTs met the inclusion criteria and focused on the subject of study. (Figure 1).

After analyzing the methodological quality of the articles via the works of Jadad AR et al. [23] and the OCEBM of Manterola CD et al. [24], four studies [15,17,28,32] obtained a score of 4 on the JADAD scale, the highest score that can be obtained in these RCTs due to the impossibility of double-blinding the studies, and only one [33] a score of 2. Regarding the OCEBM [24], the five studies [15,17,28,32,33] had a level 1b (Table 1).

**Figure 2:**
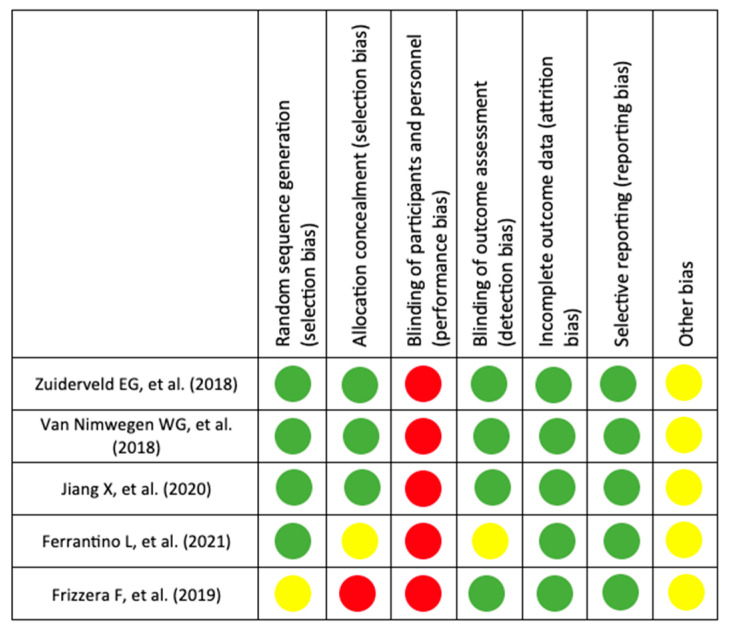
Risk of bias assessment summary [15,17,28,32,33].

Figure 2 displays the results of the risk of bias assessment. None of the trials were at low risk of bias due to the blinding of participants was impossible to achieve. Despite this, it can be considered that three trials [17,28,32] were at low risk.

Regarding the populations analyzed, two studies were carried out in the Netherlands [17,28], one in China [32], another in Brazil [33], and another in Italy [15], with all of them published between the years 2017 and 2021.

The five RCTs that were included [15,17,28,32,33] analyzed a total sample of 245 patients, with samples greater than 60 patients in 60% of the studies [15,17,28] and the smallest being 24 [33] (Table 2). There were three reported dropouts, two among the patients in the study by Jiang X et al. [32], and one among the patients in the CG of the study by Ferrantino L et al. [15]. Six implants failed, of which three were from the study group (SG) [15,17,28,33] and another three from the CG [15,17,28,33]. From the study by Van Nimwegen WG et al. [17], four patients from the CG and four patients from the SG were excluded due to irregularities in the study casts taken before and/or 12 months after the intervention.

All subjects were over 18 years of age; 43.20% males (*n* = 105) and 56.32% females (*n* = 138) were included. All the included studies [15,17,28,32] divided the sample into two groups, the SG with II and CTG and the CG with II and no CTG, except for one study [33] that divided the sample into three groups, adding a group that received a Mucograft^®^ collagen matrix. In four of the studies [15,28,32,33], the CTG was performed simultaneously with the II, unlike one study [17] that did not mention the timing of the CTG placement.

In two studies [17,28], grafts were obtained from the tuberosity, in two [32,33] from the palate, and in one [15], grafts were obtained from both areas. In two of the studies [15,28], the missing teeth replaced with an IIP were from the aesthetic maxillary area (incisor, canine, or first premolar); in another article [15], implants from the aesthetic lower sector were also included, and in two articles [32,33], only implants in upper incisors were included.

On the other hand, in 80% of the studies, antibiotic prophylaxis was indicated; in two of them [17,28], with Amoxicillin 500 mg or Clindamycin 300 mg starting 1 day before surgery and for 7 days in total; in another study [32], with Cefuroxime 0.25 g 1 h before surgery; and the last one [15] with 2 g of Amoxicillin 1 h before surgery, using 600 mg of Clindamycin in allergic patients. One study did not indicate prophylaxis [33], but postoperative antibiotic therapy was prescribed with Amoxicillin 500 mg for 7 days.

Regarding the position and torque of the II, these were slightly different in the studies analyzed. Overall, 120 implants [17,28] were placed 3 mm apical to the cementoenamel line of the adjacent tooth, 66 implants [32,33] were placed 4 mm apical to the gingival margin, and 59 implants [15] were placed between 1 mm and 2 mm below the buccal bone crest. An amount of 60 implants [17] were inserted at ≥45 Newtons (Nw) of torque, 101 implants [15,32] were inserted at >35 Nw, 24 implants [33] at 32 Nw, and in 60 implants [28], this parameter was not mentioned.

All the studies agreed on filling the gap between the implant and the vestibular cortex. In 41.2% (*n* = 101) [15,32], the gap was filled with xenograft; in 48.9% (*n* = 120) [17,28], a 1:1 mixture of xenograft and autologous bone, and in 9.7% (*n* = 24) [33] was filled with xenograft and a collagen membrane (Bio-Gide^®^; Geistlich Pharma AG, Wolhusen, Switzerland) placed internally in contact with ST.

All authors [15,17,28,32,33] provisionalized the IIP on the day of surgery, screwing the provisional crown to 20 Nw in 84 implants [28,33], leaving the crown free of contact with the antagonist. Definitive rehabilitation was performed after 3 months in 48.9% (*n* = 120) [17,28] and after 6 months in the other 51% (*n* = 125) [15,32,33] using a screw-retained crown or cemented.

A percentage of 86.2% (*n* = 263) of the studies had a 12-month follow-up of 82.8% (*n* = 203) [15,17,28,33] and in 17.1% (*n* = 42), a 6-month follow-up [32].

The parameters PES, ICAI, and CTG were analyzed for the assessment of the esthetic results. The success of the CTG on II differs slightly between studies, which made the comparison between them difficult. The gum level is analyzed in most of the studies as a primary parameter, with the measurement of the vestibular midpoint. This parameter is reported in 80% of them [17,28,33], obtaining better results, but without statistically significant differences, in the groups with IIP and CTG. Regarding the thickness of the vestibular ST, a parameter reported in 80% of the studies [17,28,33], all the studies found differences in volume between the SG and the CG. In three studies [28,32,33], the group with CTG obtained better results than those without CTG, reporting that the biotype and the use of CTG significantly influence bone loss. In another study [17], both groups presented a loss of vestibular ST thickness, but it was slightly higher in the SG and was not statistically significant. Marginal bone loss was analyzed in 40% of the studies [17,28]; one study [28] showed marginal bone loss in the mesial of the implant and a distal gain in both groups; another one [17] showed bone loss in the CG and bone gain in the SG. The aesthetic result was mentioned in 66.66% of the studies [15,17,28,33], and better results were obtained for the SG in two of them [15,33]; another study [17] showed better statistically significant outcomes in favor of the CG in terms of peri-implant ST texture but in favor of the SG in terms of gingival margin level. In the other study [28], 78.1% acceptance of the cases was reported regarding the peri-implant mucosa, and 85.1% of the cases regarding the crown of the implant. One study [32] analyzed patient satisfaction, finding no significant differences between groups.

The secondary variables of the studies also differ between them, finding the analysis of parameters such as probing, plaque index, bleeding, gingival index, thickness of keratinized gingiva, papilla volume, survival, and success of the implant, apart from the apico-coronal or vestibule–lingual position of the implant.

In 60% of the studies [17,28,33], the results were shown in favor of CTG with immediate implantation with an immediate provisional crown, while in 40% of the studies [15,32], no statistically significant differences were found between performing CTG or not.

**Figure 3:**
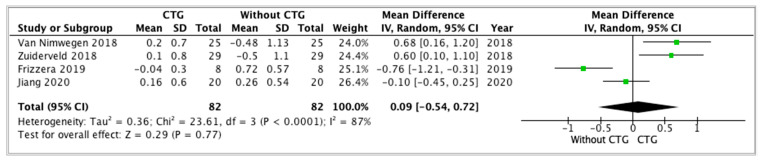
Forest plot. Studies evaluating buccal gingiva level by adding a connective tissue graft (simultaneous or delayed) vs. no graft at immediate implant placement. [17,28,32,33].

The variables analyzed by the studies showed heterogeneous values and were difficult to compare between studies. Four studies [17,28,32,33] evaluated the differences between the vestibular gingiva levels. Forest plots were produced to graphically represent the differences in the buccal gingiva midpoint when placing II with CTG and without CTG (Figure 3). A mean difference in vestibular gingiva level of 0.09 mm and a *p*-value = 0.05 were statistically not significant (95% CI: −0.54 to 0.72, *p* = 0.05). The heterogeneity between studies was high, showing an I^2^ =87% (*p* < 0.0001). Thus, statistically not significant results were found to be in favor of adding a CTG to the II, but with a favorable trend for CTG.

## 4. Discussion

This systematic review compared the benefits in terms of gingival health and aesthetics between adding a CTG (simultaneous or delayed) in IIP and cases in which no graft was performed.

IIP provides advantages in terms of treatment time and the preservation of ST morphology if the implant is performed simultaneously with its provisionalization, with 100% success rates [34]. Although some authors, such as Cosyn J et al. [35], continued to report success rates of 94.9% for II, these were lower than the success rates (98.9%) for delayed implants. In our review, a success rate of 97.39% for II was obtained.

Many factors are involved in the aesthetic result, such as the type of tooth, gingival phenotype, position of the ST margin [15], the tridimensional position of the implant, and the emergence profile. The depth of the implant, the interproximal and coronal position, and the axial inclination are also elements to consider [36]. In the RCTs included in our review [15,17,28,32,33], the insertion depth of the implant varies slightly. Once placed, the prosthetic decision to select the emergency shape of the abutment and the material was fundamental and conditioned by the position of the implant [36]. Steigmann M et al. [37] correlated the design of the emergence profile with the position of the implant, creating a convex emergence profile when the implant was positioned lingually to press ST, slightly concave when the implant was centered and concave when placed slightly buccal to increase ST thickness. On the other hand, Frizzera F et al. [33] focused on the subgingival contour, stating that a palatal position of the implant and the fabrication of a provisional with a concave subgingival contour allowed the creation of an internal void between the gingival margin and the immediate provisional and improved the ST thickness when CTG was not performed [33].

However, in our review, in all the RCTs, immediate provisionalization was performed on the same day of surgery. According to Ferrantino L et al. [15], IIP reduces midfacial recession by 0.75 mm. Previously, da Rosa JC et al. [38], demonstrated high clinical predictability and good cosmetic results if the extraction socket is intact or even in the presence of a dehiscence of the vestibular cortex. According to Jiang X et al. [32], provisionalization achieved favorable cosmetic results in recent clinical studies as long as the implant is placed in a correct three-dimensional position, a bone graft is applied, and CTG is placed.

In the study by Nimwegen WG et al. [17], it was reported that CTG with provisionalization did not show less gingival volume loss at 12 months compared to provisionalization without CTG. Hassani A et al. [39] analyzed the IIP at a maximum of 48 h versus deferred at 3 months, concluding that there were no significant differences in marginal bone loss or in pink aesthetics.

Thus, a CTG can compensate for the loss of ST in the buccal area, but it cannot help maintain the buccal gingival margin, and neither can it change the patient’s gingival biotype [15,32]. Supporting these statements, previous studies [18,40,41] showed better preservation of the vestibular gingiva when a CTG was applied simultaneously with an immediate provisional implant. On the other hand, in the study by Jiang X et al. [32], no statistically significant differences were observed between groups in terms of gingival recession. In addition, it must be considered that CTG has greater morbidity for the patient, requiring a donor site, greater surgeon skill, and sometimes a painful postoperative period [15].

The number of IIPs with immediate provisionalization has increased in recent years, thus minimizing the duration of treatment, the number of interventions, and obtaining good aesthetic results [42]. However, an IIP is not capable of avoiding hard remodeling and ST that occurs after an extraction. Thus, the recession that occurs in the vestibular face of the alveolus is the most frequent complication after the placement of an IIP [42]. According to the review by Chen S et al. [43], more than 26% of the IIP presented a recession in the buccal gingiva ≥ 1 mm. Some of the factors that influenced this recession were the presence of a thin vestibular cortex (<1 mm) or a defect thereof [32], an implant in a vestibular position, and a fine biotype [28]. Regarding the thickness of the vestibular cortical bone, if it presents a thickness between <0.5 and 1 mm, this bone will be more susceptible to greater reabsorption and greater ST recession [43]. On the other hand, according to our review, authors such as Zuiderveld EG et al. [18] did not find significant levels of correlation between the level of the vestibular gingiva and the thickness of the vestibular cortex. To prevent the loss of this cortical bone, some authors suggest the placement of a bone graft in the gap between the cortical bone and the implant [44]. A gap of at least 2 mm should be filled with bone graft to create more peri-implant hard tissue in the vestibular area so that the gingival biotype does not influence the risk of a recession. In our review, the size of the gap was not mentioned, but in all the studies, the authors refer to filling it with bone graft [18,28]. Authors such as Van Nimwegen WG et al. [17] reported that in recent years, the thickness of the post-extraction vestibular cortical bone had been considered a risk factor for ST alterations. A thin vestibular cortex (<1 mm), made up practically of hard bone, would undergo resorption regardless of the type of treatment followed [32]. Authors such as ElAskary A et al. [45] reported that by means of the vestibular alveolus therapy technique, fewer changes in the soft and hard tissues. These results could be related to the less invasive nature of the technique and the conservation of the surrounding dento-gingival tissue complex.

In reference to the influence of the gingival biotype, there were different opinions. According to Seysenss L et al. [42], gingival biotype plays an important role in terms of ST collapse and the risk of buccal gingiva recession around II. The anterior sector is characterized by frequently presenting a thin gingival biotype; for this reason, several authors from our review use CTG to increase its thickness when an II was placed [17,29,31,34]. On the other hand, other authors, like Zuiderveld EG et al. [28], reported that the gingival biotype did not seem to be a predisposing factor for changes at the marginal bone level. This variety of opinions could be explained by the different methods applied to distinguish between thick and fine biotypes [28], but it seems to be clear that ST contraction can compromise aesthetic results due to gingival involvement, especially in the upper jaw [15].

Jiang X et al. [32] reported that systematic reviews had shown that II could lead to advanced gingival recession (>1 mm), which could result in aesthetic failure of the implant restoration. In our review, there were different positions. Authors such as Zuiderveld EG et al. [18] reported no significant differences between groups in terms of aesthetic levels; but Van Nimwegen WG et al. [17] reported better results than the CG in pink aesthetic levels (PES), although without statistically significant results. Regarding the ST texture, the results were also significant and better in the CG, resulting in greater deformation of the mucosa and healing of the peri-implant ST [17].

About the donor side, Rojo E et al. [5] did not find significant differences. Concluded that grafts from the tuberosity and the lateral area of the palate could obtain similar results, although showing a favorable trend to tuberosity grafts in terms of CTG thickness, keratinized gingiva width, and PES result.

In general, the studies in this review reported greater vestibular gingival recession when CTG was not applied. In the present work, it was possible to perform a meta-analysis with 4 RCTs [17,28,32,33] which included 164 immediate implantations (implants with CTG: 82, implants without CTG: 82). These articles [17,28] reported less advancement of the gingiva apically when a CTG was applied, with the exception of studies by Jiang X et al. [32] and Frizzera et al. [33], which reported better results without applying CTG.

The lower ST gained from the grafts extracted from the palate can be attributed, as shown in the study by Jiang X et al. [32] and Frizzera et al. [33] due to being composed of more glandular and adipose tissue than tuberosity grafts as reported by authors such as Sanz-Martín I et al. [46]. Instead, the other studies [17,28] obtained the CTG of the tuberosity.

Our meta-analysis is not statistically significant, but it is similar to the results obtained in the meta-analysis carried out by Seeyssens L et al. [42] published in 2020, where they found a greater buccal gingiva midpoint distance when CTG was not used and claimed to be clinically relevant results as the risk of asymmetry ≥ 1 mm at the mid-vertical ST level was 12 times lower after immediate implantation with CTG compared to the group without CTG. Likewise, in the meta-analysis by Aldhohrah T et al. [6], significant results were obtained in favor of the CTG group.

The follow-up time is a factor to consider when analyzing the studies. ST changes continue after one year; recent literature shows a more pronounced ST recession at 5 years [28]. Van Nimwegen WG et al. [17] did not show significant differences between the CTG and non-CTG groups; however, at a 12-month follow-up, the CG showed a greater loss of mucosal volume. This loss was related to factors such as the physiological resorption of the vestibular cortex after extraction and IIP and the surgical technique used to place the CTG in the SG, inducing additional bone loss by cutting the vascularization from the mucosa to the vestibular cortex [18]. The short-term results obtained by Zuiderveld EG et al. [18] showed how the CTG gave greater vestibular bone loss and less vestibular gum recession than when CTG was not placed. Concluded that the decrease in the thickness of the vestibular cortex was not accompanied by a greater recession in the vestibular gingiva when applying the CTG. Hence, it is assumed that the CTG can limit the recession [18].

Our review had limitations despite the fact that we found many published studies dealing with CTG, some of which compared the placement of CTG at the time of implant placement and without CTG. However, there were few studies that met the inclusion requirements in our review. Mainly, the heterogenicity between the studies is high (87%). Most of the published RCTs have a short follow-up period of at most 12 months, so studies with a longer follow-up period were required to observe recessions. In addition, the parameters and the methods analyzed were heterogeneous, a fact that made it difficult to compare them. Future RCTs should involve large populations and long-term follow-ups to analyze the influence that these variables have on the outcomes.

## 5. Conclusions

Despite the low evidence and the limitations of the study, the connective tissue graft associated with the immediate implant, it is possible to maintain the gingival level, but not increase the volume, regardless of the gingival biotype. The use of connective tissue is favorable for achieving successful cosmetic results when immediate implant placement with a provisional prosthesis is planned. With the placement of an immediate implant, bone resorption is not avoided, and to preserve the vestibular cortex (a key parameter in the aesthetics of the implant), its optimal placement is of great importance. These results may allow clinicians to adopt new forms of treatment to achieve better results.

## Figures and Tables

**Table 1:** Comparative summary of the analysis of the methodological quality according to JADAD [23] and the levels of evidence according to Oxford (OCEBM) [24] of the included studies.

Author, Year	JADAD						Level of Evidence (OCEBM)
	Randomization	Adequate Randomization?	Double-Blind	Adequate Masking?	Description of Dropouts	TOTAL (0–5)	
Zuiderveld EG et al. (2018) [28].	+1	+1	0	+1	+1	4	1 B
Van Nimwegen WG et al. (2018) [17].	+1	+1	0	+1	+1	4	1 B
Jiang X et al. (2020) [32].	+1	+1	0	+1	+1	4	1 B
Ferrantino L et al. (2021) [15].	+1	+1	0	+1	+1	4	1 B
Frizzera F et al. (2019) [33].	+1	−1	0	+1	+1	2	1 B

**Table 2:** Comparative summary of the articles included in the review.

Author (Year), Country. [Type of Study]	Population (Mean Age)	Surgical Procedure and Provisionalization	Parameters Evaluated and Results					Follow-Up	
			Buccal Gingiva Level (Mean ± ds)	Gum Thickness	Probing Depth (Mean ± ds)	Vestibular Cortical Thickness (Mean ± ds)	Aesthetic Result (Mean ± ds)		Conclusions
Zuiderveld EG et al. (2018), Netherlands [28]. [RCT]	60 SG: 30 (45.5 ± 15.5) CTG of the tuberosity. CG: 30 (47.8 ± 16.5) without CTG.	1:1 mixture of autologous bone from the tuberosity and xenograft to fill the gap. Provisional crown the same day of surgery, at 20 Nw, and free of contact. After 3 months, the final zirconia crown at 35 Nw.	12 m −0.5 ± 1.1 mm in the CG and 0.1 ± 0.8 mm in the SG (*p* = 0.03).		1st m SG 2.6 ± 1.4 and CG: 2.2 ± 0.9 12 m SG 2.5 ± 1.2 CG: 2.3 ± 0.9.		12 m PES total mean change at 12 months of 6.8 ± 1.5 in the CG and 6.4 ± 1.5 in the SG.	1 m and 12 m	Immediately placed and provisionalized implants lead to less recession of the peri-implant soft tissue at the mid-buccal aspect, irrespective of the gingival biotype
Van Nimwegen WG et al. (2018), Netherlands [17]. [RCT]	60 SG: 30 (45.5 ± 15.5) with CTG of the tuberosity. CG: 30 (47.8 ± 16.5) without CTG.	1:1 mixture of autologous bone and bovine xenograft to fill the gap. A provisional crown was placed on the same day of surgery, free of contact. After 3 m, a definitive zirconia crown was placed.	SG: 0.20 ± 0.70 mm CG: −0.48 ± 1.13 mm.	SG −0.68 ± 0.59 mm CG: −0.49 ± 0.54 mm	SG: 2.28 ± 0.79 mm CG: 2.44 ± 1.19 mm		SG Change in level gingival marginal 1.80 ± 0.50 Texture 1.80 ± 0.50 PES total score: 11.28 ± 1.67 CG: Change in level gingival marginal 1.44 ± 0.71 Texture 2.00 ± 0.00 PES total score: 11.36 ± 1.65	12 m	CTG cannot fully compensate for the underlying facial bone loss, although a significantly more coronally located mid-facial mucosa level was found when a CTG was performed
Frizzera F et al. (2019), Brazil [33]. [RCT]	24 SG 1: 8 collagen matrix (Mucograft Geistlich) SG 2: 8 CTG of the palate. CG: 8	Collagen membrane (bio-guide^®^) and Bovine bone with 10% porcine collagen (Bio-oss^®^) to fill the gap between the membrane and the implant. Prefabricated resin crown on the same day of surgery, at 20 N and in infra-occlusion. After 6 m, the final crown was placed.	At 12 m SG 1 −0.28 ± 2.51 mm SG 2 −0.55 ± 3.51 mm CG 0.35 ± 3.69 mm,	SG 1: Baseline of 0.98 ± 0.21 mm, at 6 m of 2.05 ± 0.41 mm, and at 12 m of 2.1 ± 0.54 SG 2: Baseline of 0.98 ± 0.29, at 6 m of 2.82 ± 0.40 and at 12 m of 3.04 ± 0.61 CG: Baseline of 1 ± 0.18, at 6 m of 2.04 ± 0.43, and at 12 m of 2.11 ± 0.60		SG 1: 1.35 ± 1.38 at 6 m 1.14 ± 1.34 at 12 m SG 2: 1.04 ± 1.01 at 6 m, 1.06 ± 0.87 at 12 m CG: 1.22 ± 0.85 at 6 m, 1.28 ± 0.92 at 12 m	SG 1: At baseline 10.63 ± 1.84 and at 12 m of 10 ± 1.3 SG 2 At baseline 9.37 ± 1.9 and at 12 m of 10.75 ± 1.38 CG 1: Baseline of 10.75 ± 2.05 and at 12 m of 9.87 ± 1.64	12 m	The use of a CTG avoided MPR and provided better contour of the alveolar ridge and greater thickness of the soft tissue at the implant facial aspect.
Jiang X et al. (2020), China [32]. [RCT]	42 SG: 21 (34.3 ± 7.0) CTG of the palate. CG: 21 (37.7 ± 13.3)	The gap was filled with DBBM xenograft (Bio-oss^®^). Provisionalization was placed in the first 24 h, without occlusal or eccentric contacts. Definitive crown 6 m after surgery.	Coordinates of gingival margin point. SG: (0.63 ± 0.53 mm, 0.16 ± 0.60 mm) at 6 m CG: (0.63 ± 0.55 mm, 0.26 ± 0.54 mm) at 6 m			The buccal plate thickness of the socket was 0.54 ± 0.20 mm for the SG and 0.69 ± 0.30 mm for the CG.		A week, 1 m and 6 m	The CTG used with IIP and provisionalization could compensate for the facial tissue collapse, but it did not benefit the maintenance of the mid-facial gingival margin position
Ferrantino L et al. (2021), Italy [15]. [RCT]	60 SG: 31 (47.68 ± 16.50) CTG of the palate or tuberosity. CG: 28 (51.07 ± 14.67)	The gap between the implant and vestibular wall was filled with xenograft (Bio-oss^®^). A customized resin crown was placed after surgery, free of occlusal contacts. The final restoration was performed 6 m after surgery.					The mean ICAi at 12 m was 4.69 for the SG and 3.45 for the CG. For the items assessing mucosa aesthetics, the SG mean was 1.77, and the CG was 2.27. For the items assessing the ICAI crown, the SG mean was 2.92, and the CG was 1.18.	A week, 1 m, 6 m, and 12 m	CTG is not mandatory to achieve successful aesthetic outcomes for a well-planned immediate implant placement with immediate non-functional provisional restoration in a fresh extraction socket.

RCT: Randomized Clinical Trial; CTG: connective tissue graft; PES: Pink Esthetic Score; ICAI: Crown Aesthetic Index; CG: Control Group; SG: Study Group; m: month; MPR: Marginal Peri-implant Recession.

## Abbreviations

ST	Soft Tissue.
IIP	Immediate Implant Placement.
CTG	Connective Tissue Graft.
RCT	Randomized Control Trial.
PRISMA	Preferred Reporting Items for Systematic Reviews and Meta-Analyses.
OCEBM	Oxford Classification of Evidence Levels.
CI	Confidence Interval.
CG	Control Group.
SG	Study Group.
PES	Pink Esthetic Score.
ICAI	Crown Aesthetic Index.
Nw	Newtons.
